# Rice-memolin, a novel peptide derived from rice bran, improves cognitive function after oral administration in mice

**DOI:** 10.1038/s41598-023-30021-3

**Published:** 2023-02-18

**Authors:** Maiko Shobako, Naohisa Shobako, Biyun Zhang, Kentaro Kaneko, Kousaku Ohinata

**Affiliations:** Division of Food Science and Biotechnology, Graduate School of Agriculture, Kyoto University, Uji, Kyoto 611-0011 Japan

**Keywords:** Peptides, Dementia

## Abstract

Many people eat polished rice, while rice bran, a by-product known to be rich in protein and expected to have potential functions for health benefits, has not been effectively utilized. In this study, we determined that orally administered Val-Tyr-Thr-Pro-Gly (VYTPG) derived from rice bran protein improved cognitive decline in mice fed a high-fat diet (HFD). It was demonstrated that VYTPG was released from model peptides corresponding to fragment sequences of original rice proteins (Os01g0941500, Os01g0872700, and allergenic protein) after treatment with thermolysin, a microorganism-derived enzyme often used in industrial scale processes. The thermolysin digest also improved cognitive decline after oral administration in mice. Because VYTPG (1.0 mg/kg) potently improved cognitive decline and is enzymatically produced from the rice bran, we named it rice-memolin. Next, we investigated the mechanisms underlying the cognitive decline improvement associated with rice-memolin. Methyllycaconitine, an antagonist for α7 nicotinic acetylcholine receptor, suppressed the rice-memolin-induced effect, suggesting that rice-memolin improved cognitive decline coupled to the acetylcholine system. Rice-memolin increased the number of 5-bromo-2’-deoxyuridine (BrdU)-positive cells and promoted the mRNA expression of EGF and FGF-2 in the hippocampus, implying that these neurotropic factors play a role in hippocampal neurogenesis after rice-memolin administration. Epidemiologic studies demonstrated that diabetes is a risk factor for dementia; therefore, we also examined the effect of rice-memolin on glucose metabolism. Rice-memolin improved glucose intolerance. In conclusion, we identified a novel rice-derived peptide that can improve cognitive decline. The mechanisms are associated with acetylcholine and hippocampal neurogenesis. Rice-memolin is the first rice-brain-derived peptide able to improve cognitive decline.

## Introduction

Rice, known as one of the three major grains, is a staple food for almost half of the world population^1^. It is usually served in the form of polished white rice; however, beneficial health effects of non-polished brown rice have also been reported^2,3^. Rice bran is well-known to be rich in protein and has a high protein efficiency ratio^4^; however, its availability remains unknown, and it is not utilized industrially. On the other hand, we have found that an enzymatic digest of bran protein exhibits an antihypertensive effect, followed by isolating novel antihypertensive peptides^5,6^. As such, the rice bran protein is a promising source of functional peptides.

The importance of Quality of Life (QOL) increases with a prolonged life expectancy. In the past 50 years, life expectancy has been prolonged globally by approximately 10 years. Therefore, there is an increasing concern over how long a human can stay healthy. Aging is known to increase inflammation in organs and induce multiple health problems such as high blood pressure, metabolic disorder, and cognitive decline, to which Western style diet might also be related^7^. In particular, cognitive decline leads to dementia and necessitates care, which has become a serious issue in countries with an aging society. Mild cognitive impairment (MCI) is a preclinical stage of dementia^8^. Completely recovering from dementia is difficult; however, it is possible to keep or to improve MCI through medical treatment or lifestyle changes such as dietary habits and exercise^9^. Especially some clinical trials of dietary intervention were previously reported^10,11^. Thus, we hypothesized that peptide derived from rice bran protein can be used to improve mild cognitive decline.

So far, epidemiological studies have revealed that glucose tolerance impairment is associated with cognitive decline^12,13^. It was reported that high-fat diet (HFD) intake, known to induce glucose intolerance, accelerates brain aging and induces cognitive decline^14–16^. Suggested causes might be brain damage, such as the reduction of proliferation and decreased numbers of immature neurons in the hippocampus^17^. We previously reported that short-term HFD intake induced cognitive decline and hippocampal dysfunction in mice^16^. In this study, we used this experimental system to examine the effect of a rice bran-derived peptide on cognitive function.

**Figure 1:**
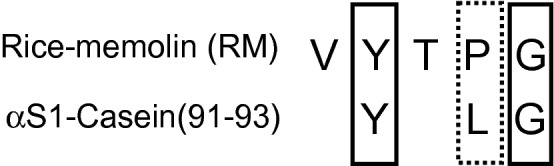
Homology of VYTPG and YLG derived from rice bran and milk casein. Solid lines indicate the same amino acid residues, dotted lines indicate similar sequences.

We previously isolated Val-Tyr-Thr-Pro-Gly (VYTPG) in the process searching for angiotensin I-converting enzyme (ACE) inhibitory peptides originated from the thermolysin digest of rice bran proteins (unpublished observation). This peptide has homology with YLG, a milk derived peptide that improved cognitive decline in the mice model^16^ (Fig. 1). We then examined the effect of VYTPG on cognitive function. VYTPG potently improved cognitive decline at a low dose compared with a known food-derived peptide after oral administration, and we termed it as rice-memolin. We also investigated the mechanisms underlying the cognitive decline improvement of rice-memolin.

## Materials and methods

### Materials

Rice bran obtained from Japonica rice (*Oryza japonica*) is commercially available in Japan, named High-Bref, was supplied by Sunbran Co., Ltd. (Tendou, Japan). VYTPG was synthesized chemically using the F-moc method and purified by reverse phase high-performance liquid chromatography (RP-HPLC). d-Glucose was purchased from Sigma-Aldrich (St Louis, MO). The plant collection and use were in accordance with all the relevant guidelines.

### Preparation of the digest of rice bran protein

Thermolysin digestion of rice bran protein was performed as described previously^5^. Briefly, 900 ml of ultrapure water and 0.36 g of thermolysin were added to 27 g of rice bran. Enzymatic digestion took place at 37 °C for 20 h. The digest was then heated for 10 min in boiling water to inactivate thermolysin. The supernatant was recovered after centrifugation (8000×*g*, 15 °C, 15 min) and its pH was adjusted to 7.0 with sodium hydroxide. The supernatant was then filtered using filter paper No.2 (Advantec, Tokyo, Japan) and freeze-dried.

Pepsin and pancreatin digests of VYTPG and LCMS analysis were performed in a similar manner to previous studies (Shobako et al.) with slight modifications to simulate peptide bioavailability. Each enzyme treatment time was 1 h.

### Digest-mixed feeding experiment

Male ddY mice (11 weeks old) were obtained from SLC (Shizuoka, Japan). They were kept in a temperature-controlled room (23 °C) on a daily 12-h light: 12-h dark cycle. After one week-acclimatization, these mice were fed MF pellets (Oriental Yeast, Tokyo, Japan) as control diet, HFD60 pellets (Oriental Yeast Co., Ltd, Japan) as high-fat diet, or HFD 60 pellets containing 0.04% rice bran protein digest (specially ordered from Oriental Yeast Co., Ltd, Japan) with free access to water for 7 days. The component of HFD60 was previously reported^18^. Animal experiments were conducted in accordance with the Fundamental Guidelines for Proper Conduct of Animal Experiment and Related Activities in Academic Research Institutions under the jurisdiction of the Ministry of Education, Culture, Sports, Science and Technology of Japan, and were approved by the Committee on Animal Experimentation at Kyoto University, Japan. All experiments were approved by the Kyoto University Ethics Committee for Animal Research Use, permission code was R3-13. All experimental procedures were performed in accordance with the ARRIVE guidelines. All efforts were made to minimize the number of animals used and to limit experimentation to what was necessary to produce reliable scientific information.

### Administration experiment

Male ddY mice (11 weeks old) were obtained from SLC (Shizuoka, Japan). They were kept in a temperature-controlled room (23 °C) on a daily 12-h light: 12-h dark cycle. After acclimatization, mice were divided into experimental groups: control diet fed group (CD group), high-fat diet fed group (HFD group), and HFD fed plus administration of the rice bran protein digest (digest group) or chemosynthetic rice-memolin group (rice-memolin group). For the CD group, a 10 kcal% fat containing diet (D12450J, Research Diets) was fed. For the HFD group, digest group, and rice-memolin group, 60 kcal% HFD (D12492, Research Diets Inc., New Brunswick, USA) was fed. Each group was fed for 7 days. For the last 3 days, the digest was administered to the digest group and rice-memolin was administered to the rice-memolin group. Mice were euthanized by cervical dislocation after the experiment. All experiments were approved by the Kyoto University Ethics Committee for Animal Research Use, permission code was R3-13. All efforts were made to minimize the number of animals used and to limit experimentation to what was necessary to produce reliable scientific information. The experiment design was referenced to our previous study^16^.

### Object recognition test and object location test

Cognitive function was evaluated on Day 6 and 7 using two behavioral pharmacological tests: novel object recognition test (ORT) and object location test (OLT). The ORT was performed as described previously in Nagai et al.^16^ with slight modification. Using the ORT, effect of donepezil, a drug currently used for the relief of cognitive deficits associated with mild to moderate Alzheimer’s diseases, was evaluated^19^. Briefly, the experimental apparatus used in this study was a square open field (50 cm × 50 cm × 50 cm) made of grey polyvinyl chloride. Two typical objects were used: wooden block and falcon tissue culture flask filled with sand. The ORT comprised three sessions: a habituation session, a familiarization session, and a test session. The habituation session was carried out on Day 6. Each mouse was placed into the experimental apparatus without any objects for 5 min. The familiarization session was performed 24 h after the habituation period. In the familiarization session, each mouse was then placed into the open field in the presence of identical two objects and allowed to explore freely until 20 s of total exploration time was reached. Exploration of the objects was considered positive when the animals' nose was facing the objects less than 1 cm away from the object. After 1 h, the test session was performed. At the test session, one object of each pair was replaced with a novel object. Each mouse was again placed into the open field for 20 s of total exploration time, and the exploration time spent on the familiar object and novel object were measured.

### BrdU incorporation in the hippocampus

BrdU incorporation was assessed as reported previously in Nagai et al. and Yamamoto et al.^16,20^ On Day 6, BrdU (100 mg/kg; Sigma Aldrich) was administered intraperitoneally. Twenty-four hours after BrdU administration, mice were euthanized and transcardially perfused with phosphate-buffered saline (PBS) followed by 4% paraformaldehyde under anesthesia (n = 4). Brains were postfixed overnight in 4% paraformaldehyde at room temperature and dehydrated in 20% sucrose (4 °C for 1 day). Serial sections of the brains were cut (30-mm sections) on a freezing microtome. After the sections were washed with PBS containing Triton X-100 (PBST), they were incubated in 2 N HCl for 45 min and washed with PBST. After blocking in 3% normal donkey serum–PBST for 1 h, sections were incubated overnight with anti-rat BrdU (1:1,000; Abcam, Cambridge, United Kingdom) and DAPI (1:100,000; Thermo Fisher Scientific, Waltham, MA, USA) at room temperature. They were incubated with the secondary antibody (1:500, donkey anti-rat Alexa 594; Thermo Fisher Scientific) for 2 h. BrdU-positive cells were counted in the hippocampus using an Olympus microscope (Olympus, Tokyo, Japan).

### RNA preparation and quantitative RT-PCR

The hippocampus was excised. Total RNA was extracted from the hippocampus using a RNeasy Lipid Tissue Kit (QIAGEN Sciences Inc.) and transcribed into cDNA with random primers using Takara PrimeScript RT Master Mix (Takara, Osaka, Japan). For quantitative PCR, we amplified the cDNA using a LightCycler 96 System (Roche Diagnostics Co., Mannheim, Germany) with THUNDERBIRD qPCR Mix (Toyobo Co., Osaka, Japan). Primer sets specific for mouse brain-derived neurotrophic factor (BDNF), nerve growth factor (NGF), neurotrophin-3 (NT-3), glial cell line-derived neurotrophic factor (GDNF), epidermal growth factor (EGF), ciliary neurotrophic factor (CNTF), fibroblast growth factor 2 (FGF2), insulin-like growth factor 2 (IGF2), and vascular endothelial growth factor (VEGF) were prepared in accordance with the manufacturer's instructions. The primer sequences are shown in Supplemental Table S1. The reactions were cycled 45 times with denaturation at 95 °C for 10 s, and with annealing and elongation at 65 °C for 60 s each. The relative expression level of each mRNA was normalized using the mRNA level of β-actin.

### Oral glucose tolerance test

Glucose lowering effect of the peptide was assessed using the oral glucose tolerance test (OGTT). The OGTT was performed as reported previously in Ogiwara et al.^21^. Briefly, glucose solution was administered orally at a dose of 2 g/kg to mice fasted for 18 h. Blood was obtained from the tail vein. The blood glucose levels were immediately measured before and 15, 30, 60, and 90 min after glucose administration using a FreeStyle Freedom (Nipro Corp., Osaka Japan). Rice-memolin dissolved in saline was administered p.o. 2 h before glucose administration. The GTT was started from noon during the light phase of the light/dark cycle.

### Data analysis

All values are expressed as the means ± SEM. To assess differences among more than three groups, analysis of variance (ANOVA) followed by post-hoc test was performed. Dunnett’ test was used for comparing the HFD group with other groups. P-values less than 0.05 were considered significant.

## Results

### Rice bran protein-originated pentapeptide VYTPG improved HFD-induced cognitive decline after oral administration

To investigate the effect of rice bran peptide on cognitive function, we performed two behavior tests: the novel ORT and the OLT. Chronic intake of HFD was reported by Lindqvist et al.^22^ to reduce cognitive function; however, we have recently found that short-term HFD intake has similar effects^16^. Thus, we used mice treated with HFD for 1 week.

**Figure 2:**
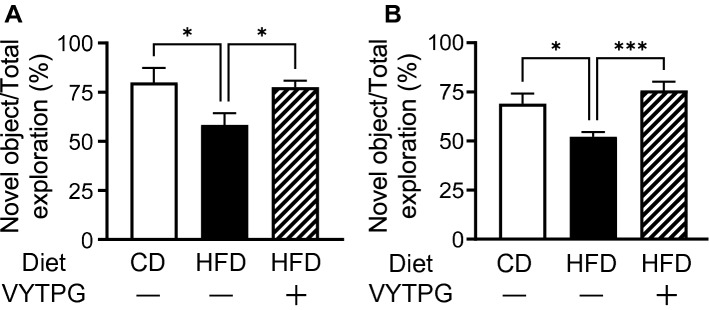
VYTPG improved cognitive decline in mice treated with HFD after oral administration. Mice were fed CD or HFD for a week and orally administered saline or VYTPG at a dose of 1 mg/kg (once a day for 3 days). (**A**) Thereafter, the novel object recognition test (ORT) was performed. (**B**) The object location test (OLT) was also performed similarly to the novel ORT. All values are means ± SEM (**A**, n = 4–6; **B**, n = 5–7). *P < 0.05, ***P < 0.001 vs. HFD group.

The results related to cognitive function are shown in Fig. 2. In the novel ORT, the approach time to new objects of mice fed HFD decreased compared with that of mice fed the control diet (CD), consistent with previous report (Fig. 2A). In contrast, orally administered VYTPG, a pentapeptide originating from rice bran protein, increased the approach time to the new object. These results suggest that VYTPG improves cognitive decline in mice fed with HFD. In the OLT, VYTPG also increased the approach time to the object placed in a new location (Fig. 2B). Thus, we demonstrated that VYTPG improved cognitive decline in two different paradigms. The effective dose of VYTPG was 1.0 mg/kg/day, and it was administrated for 3 consecutive days, which is 1/10 of the effective dose of previously reported for YLG (10 mg/kg/day for 1 week). To the best of our knowledge, VYTPG is the most potent peptide for improving cognitive decline even after oral administration.

### VYTPG, named rice-memolin, is enzymatically released from rice bran proteins

**Figure 3:**
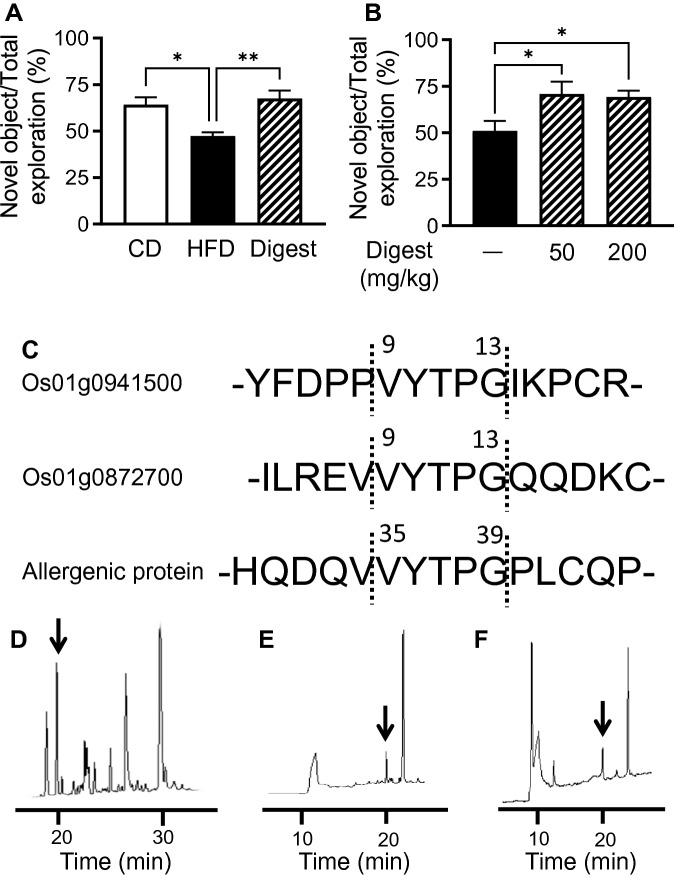
Effect of rice bran digest on cognitive decline and release of VYTPG, named as rice-memolin, from three model peptides corresponding to partial sequences of the original proteins. (**A**) Mice were fed CD, HFD, or 0.04% digest-mixed HFD for a week. Thereafter, the novel ORT was performed. (**B**) Mice were fed HFD for a week and the digest was administered orally at a dose of 50 and 200 mg/kg (once a day for 3 days). Thereafter, the novel ORT was performed. All values are means ± SEM (**A**, n = 5–8; **B**, n = 12–13). *P < 0.05, **P < 0.01 vs. HFD group. (**C**) Each model peptide, corresponding to partial sequence of rice protein candidate containing VYTPG sequence, was synthesized. These HPLC chromatograms showed that VYTPG was released by thermolysin digestion from the model peptides corresponding to the partial sequence of (**D**) Os01g0941500, (**E**) Os01g0872700, and (**F**) allergenic protein.

VYTPG was detected and isolated from the thermolysin digest by RP-HPLC. To assess its origin, the amino acid sequence of rice proteins including VYTPG was searched using BLAST online (https://blast.ncbi.nlm.nih.gov/Blast.cgi). We found that VYTPG corresponded to Os01g0941500(9–13), Os01g0872700(9–13), and allergenic protein(35–39) (Fig. 3C). Model peptides, a sub-sequence of Os01g0941500, YFDPPVYTPGIKPCR; Os01g0872700, ILREVVYTPGQQDKC, and allergenic protein, HQDQVVYTPGPLCQP were synthesized using the F-moc method. VYTPG was found in the thermolysin-digested model peptide (Fig. 3D–F). On the other hand, VYTPG itself existed even after digestion by pepsin and pancreatin present in the gastrointestinal tract digestion (Supplemental Fig. S1). These results suggest that VYTPG was resistant against gut proteases.

The effect of the rice bran digest on cognitive function was also tested using the novel ORT. The thermolysin digest increased the approach time to the novel object compared with that of the HFD group (Fig. 3A,B), suggesting that the digest also ameliorated cognitive decline which is similar to VYTPG.

Taken together, VYTPG potently improved cognitive decline, and this peptide was demonstrated to be enzymatically produced from rice bran proteins. We then named it rice-memolin.

### Cognitive improving effect of rice-memolin was associated with acetylcholine

**Figure 4:**
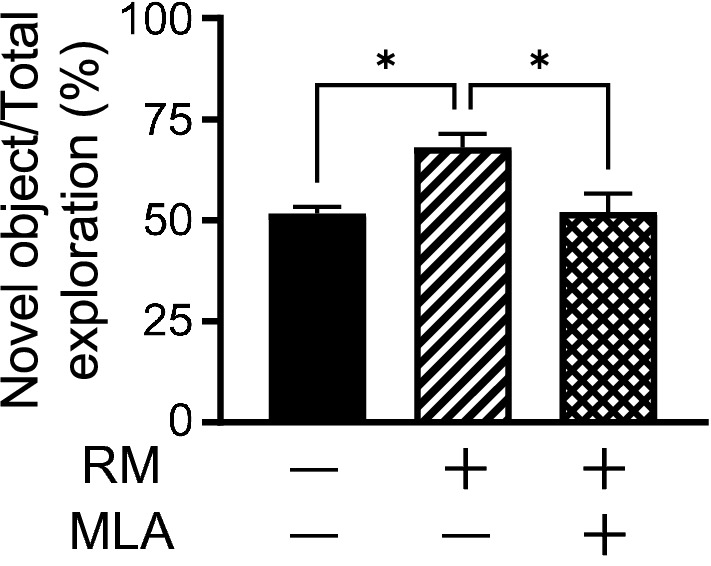
Rice-memolin improves cognitive decline depends on the acetylcholine system. Mice were fed HFD for a week and rice-memolin was administered orally at a dose of 1 mg/kg (once a day for 3 days) with or without administration of methyllycaconitin (MLA), an antagonist for α7 nicotinic acetylcholine receptor, at a dose of 0.3 mg/kg. Thereafter, the novel ORT was performed. All values are means ± SEM (n = 3–5). *P < 0.05, **P < 0.01, ***P < 0.001 vs. HFD group. RM: Rice-memolin.

Acetylcholine is a transmitter associated with memory and learning. The α7 nicotinic acetylcholine receptor (α7nAChR) is also present in the brain, especially in the cerebrum and hippocampus, and is associated with memory and learning^23^. To investigate the mechanism of rice-memolin, we performed a novel ORT using methyllycaconitine (MLA; Sigma Aldrich), an antagonist of α7nAChR. Injection of MLA (0.3 mg/kg, i.p.) inhibited the effects of rice-memolin (Fig. 4), suggesting that rice-memolin improves cognitive decline via α7nAChR.

### Rice-memolin increased neurogenesis and expression of neurotrophic factors in the hippocampus

**Figure 5:**
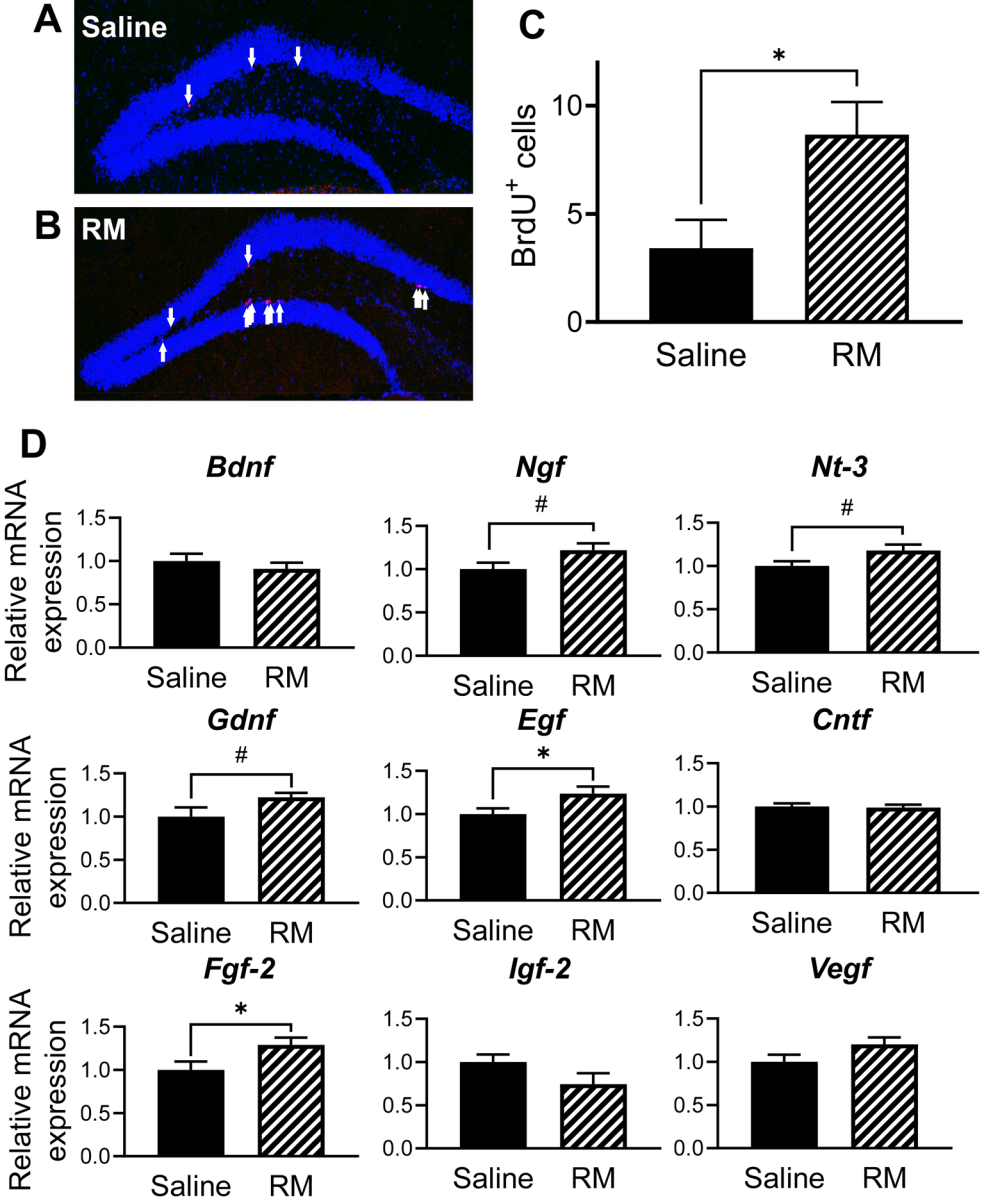
Rice-memolin increases hippocampal neurogenesis and mRNA expression of neurotrophic factors in mice fed with HFD. Representative histology of the mice administered (**A**) saline or (**B**) rice-memolin and (**C**) the number of BrdU-positive cells. (**D**) Changes in the hippocampal mRNA expression of neurotrophic factors. All values are means ± SEM (**A**–**C**, n = 4; **D**, n = 6). ^#^*P* < 0.1, *P < 0.05 vs. saline group. RM: Rice-memolin.

To investigate the effect of rice-memolin on neurogenesis in the hippocampus, hippocampal BrdU-positive cells were examined. We reported previously that 1 week of HFD intake reduced BrdU-positive cells in the hippocampus^16^. BrdU-positive cells in the hippocampus significantly increased after injection of rice-memolin (Fig. 5A–C), suggesting that rice-memolin increased hippocampal neurogenesis that was depressed by HFD intake.

Neurotrophic factors are associated with hippocampal neurogenesis and cognitive function. We examined the hippocampal mRNA expression of neurotrophic factors, including *BDNF*, *NGF*, *NT-3*, *GDNF*, *EGF*, *CNTF*, *FGF-2*, *IGF-2*, and *VEGF*. Administration of rice-memolin significantly increased the mRNA expression of EGF and FGF-2, and slightly increased NGF, NT-3, and GDNF (Fig. 5D). This suggests that changes in neurotrophic factors might contribute to neurogenesis and cognitive function.

### Rice-memolin reduces blood glucose

**Figure 6:**
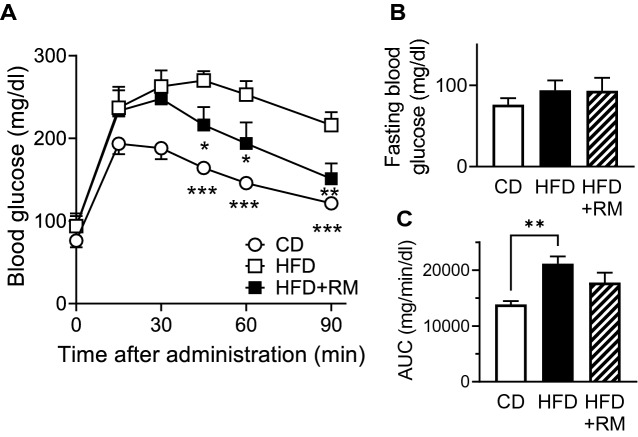
Rice-memolin reduces blood glucose. (**A**–**C**) The OGTT was performed, n = 6–7. *P < 0.05, **P < 0.01, ***P < 0.001 vs. HFD group. RM: Rice-memolin.

Impaired glucose tolerance is a factor of cognitive decline^12,13^. We then performed an OGTT, as shown in Fig. 6A–C. Fasting-glucose levels were not different among all groups (Fig. 6B). HFD intake increased the blood glucose level, and rice-memolin ameliorated such increase (Fig. 6A,C).

## Discussion

In this study, we found rice-memolin (VYTPG), a novel rice bran-derived peptide to improve cognitive decline. Rice-memolin potently ameliorated cognitive decline induced by short-term HFD intake after oral administration. Several protease-digested rice bran showed functional properties such as hypotensive^24^, anti-diabetic^25^ and anti-inflammatory^26^ effects. However, a few studies have isolated the functional amino acid sequence^27^. This is the first rice bran-derived peptide to improve cognitive decline.

Aging reduces hippocampal neurogenesis and synaptic plasticity through multiple factors, including structural alternations such as increasing altered intracellular signaling, gene expression, and neuroinflammation^28^. Such hippocampal dysfunction leads to cognitive decline. Aging also induces decreases in acetylcholine in brain regions, including the hippocampus, and weakens cholinergic signaling, thereby suppressing cognitive function^29,30^. For example, the acetylcholine esterase donepezil, which prevents the degradation of acetylcholine and promotes cholinergic neurotransmission, has been widely used to treat dementia. This study suggested that rice-memolin, a peptide contained in the enzymatic digest of rice bran protein, improves cognitive decline by stimulating acetylcholine receptor α7nAChR. Acetylcholine stimulation increased hippocampal neurogenesis^31^. We also noted an increase in hippocampal neurogenesis by rice-memolin, which may be related to increased acetylcholine signaling. Rice-memolin may be a novel dementia mediation or preventive agent.

It was reported that neurogenesis occurs throughout life even in the adult brain, especially in the hippocampus^32,33^. We also uncovered that short-term HFD intake suppresses hippocampal neurogenesis and mRNA expression of neurotrophic factors, resulting in cognitive decline, as reported previously^16,34^. On the other hand, administration of rice-memolin promoted hippocampal neurogenesis, increased mRNA expression of EGF and FGF-2, and slightly increased NGF, NT-3, and GDNF. NGF is a neurotrophic factor produced and secreted by glial cells^35–37^ that plays a role in the maintenance and survival of cholinergic neurons releasing acetylcholine. GDNF is also produced and secreted from glial cells, and it functions in the maintenance and survival of dopaminergic neurons^38,39^. Both acetylcholine and dopamine are neurotransmitters involved in memory^39–41^. NT-3 is a neurotrophin that promotes the survival and differentiation of neural progenitor cells^42,43^. Both FGF-2 and EGF are essential for the proliferation and survival of neural precursor cells^44,45^, and hippocampal neurogenesis depends greatly on FGF-2^46^. An enriched environment, including progenitor cell proliferation and cell survival, improves cognitive performance^47,48^. Orally administered rice-memolin may alter the hippocampal expression of these genes, thereby affecting neurogenesis and improving cognitive function.

Brain inflammation is thought to play a key role in HFD-induced cognitive loss^14^, whereas rice-memolin had no effect on the hippocampal mRNA expression of inflammatory cytokines such as tumor necrosis factor-α, interleukin (IL)-1α, IL-1β, and IL-6 (Supplementary Table S2). Rice-memolin might improve HFD-induced cognitive decline independent of the inflammatory response.

Insulin resistance also induces cognitive dysfunction, and glucose intolerance was suggested to be a risk factor for dementia in a comparative study^12^. Others reported that activation of AMPK in skeletal muscle improved cognitive disorder^49^. We found that rice-memolin improved glucose tolerance, suggesting that the cognition-improving effects of rice-memolin are associated with not only an increase in acetylcholine and hippocampal function but also improved glucose tolerance. Further studies will reveal the mechanisms underlying peptide-induced improvement of glucose metabolism, including the involvement of muscle AMPK activation.

**Figure 7:**
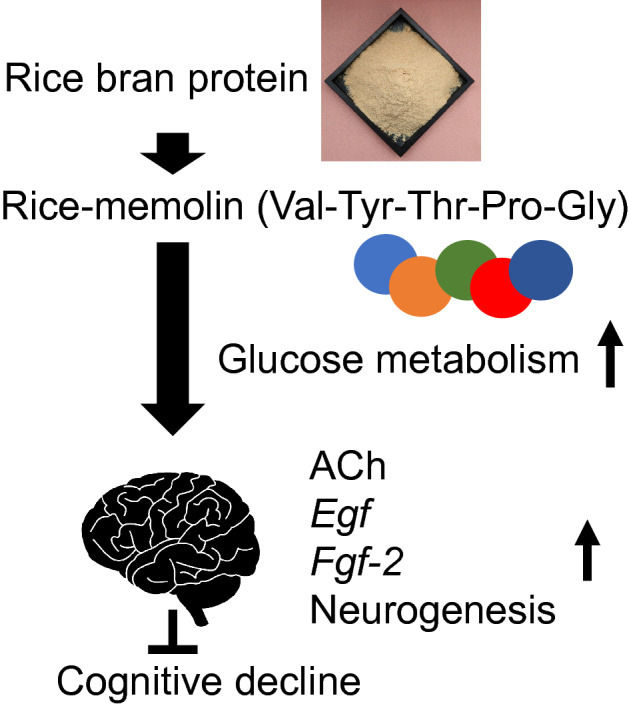
Putative scheme on the mechanism underlying cognitive decline improvement by rice-memolin derived from rice bran protein.

Several food-derived peptides have been reported to improve cognitive function^16,50,51^; however, higher concentrations and more frequent administration than rice-memolin were required. On the other hand, prevention of cognitive decline by processed rice bran was reported, but functional substances were not cleared and some of them were considered as γ-oryzanol^52–55^. Rice-memolin is orally active and the most potent cognition-improving peptide derived from food to the best of our knowledge. Cognition-improving molecules may be taken daily as an easy-to-eat food to prevent cognitive impairment at an early stage. To sum up, a schematic diagram of rice-memolin was summarized in Fig. 7.

Other limitations are still existing. Since we tested only a diet related cognition decline model, evaluation of other models such as aged mouse model is needed. The cognitive improvement of rice bran protein digest appears to be saturated at 50 mg/kg (Fig. 3b), but further dose-dependent studies are needed. Evaluation in human trials, especially considering the relation between aging and diets might also be needed. Further studies on metabolism of rice-memolin, especially in the liver, and on its half-life are required, while the safety of the rice bran digest containing this peptide had been checked by human clinical study^56^.

In conclusion, we found VYTPG named as rice-memolin, a novel pentapeptide derived from rice bran protein, improved cognitive decline in mice. This improvement might be coupled to the acetylcholine system. Rice-memolin tends to induce hippocampal neurogenesis and improve glucose metabolism. We also demonstrated that rice-memolin is enzymatically released from rice bran proteins using model peptides corresponding to partial sequences of known rice bran proteins.

## Supplementary Information

Supplementary Information.

## Data Availability

The datasets used and/or analysed during the current study available from the corresponding author on reasonable request.
