# Supplementary material for: Rice-memolin, a novel peptide derived from rice bran, improves cognitive function after oral administration in mice
Source: Sci Rep. 2023 Feb 18;13:2887. doi: 10.1038/s41598-023-30021-3 (PMC9938899; doi:10.1038/s41598-023-30021-3)
Supplement: Supplementary file 1 — Supplementary Information. [file 41598_2023_30021_MOESM1_ESM.pptx]

## Slide 1
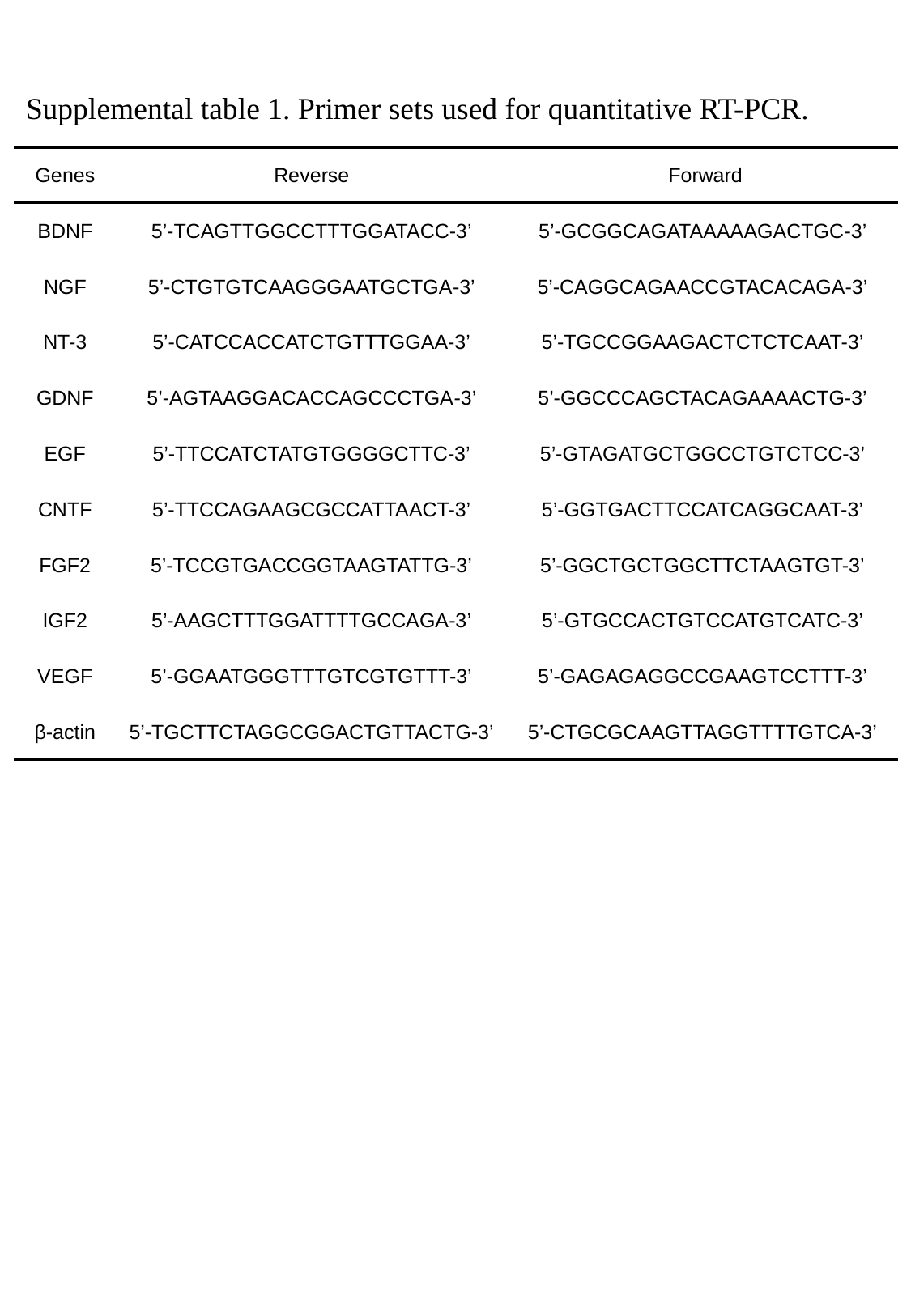

Supplemental table 1. Primer sets used for quantitative RT-PCR.
| Genes | Reverse | Forward |
| --- | --- | --- |
| BDNF | 5’-TCAGTTGGCCTTTGGATACC-3’ | 5’-GCGGCAGATAAAAAGACTGC-3’ |
| NGF | 5’-CTGTGTCAAGGGAATGCTGA-3’ | 5’-CAGGCAGAACCGTACACAGA-3’ |
| NT-3 | 5’-CATCCACCATCTGTTTGGAA-3’ | 5’-TGCCGGAAGACTCTCTCAAT-3’ |
| GDNF | 5’-AGTAAGGACACCAGCCCTGA-3’ | 5’-GGCCCAGCTACAGAAAACTG-3’ |
| EGF | 5’-TTCCATCTATGTGGGGCTTC-3’ | 5’-GTAGATGCTGGCCTGTCTCC-3’ |
| CNTF | 5’-TTCCAGAAGCGCCATTAACT-3’ | 5’-GGTGACTTCCATCAGGCAAT-3’ |
| FGF2 | 5’-TCCGTGACCGGTAAGTATTG-3’ | 5’-GGCTGCTGGCTTCTAAGTGT-3’ |
| IGF2 | 5’-AAGCTTTGGATTTTGCCAGA-3’ | 5’-GTGCCACTGTCCATGTCATC-3’ |
| VEGF | 5’-GGAATGGGTTTGTCGTGTTT-3’ | 5’-GAGAGAGGCCGAAGTCCTTT-3’ |
| β-actin | 5’-TGCTTCTAGGCGGACTGTTACTG-3’ | 5’-CTGCGCAAGTTAGGTTTTGTCA-3’ |

## Slide 2
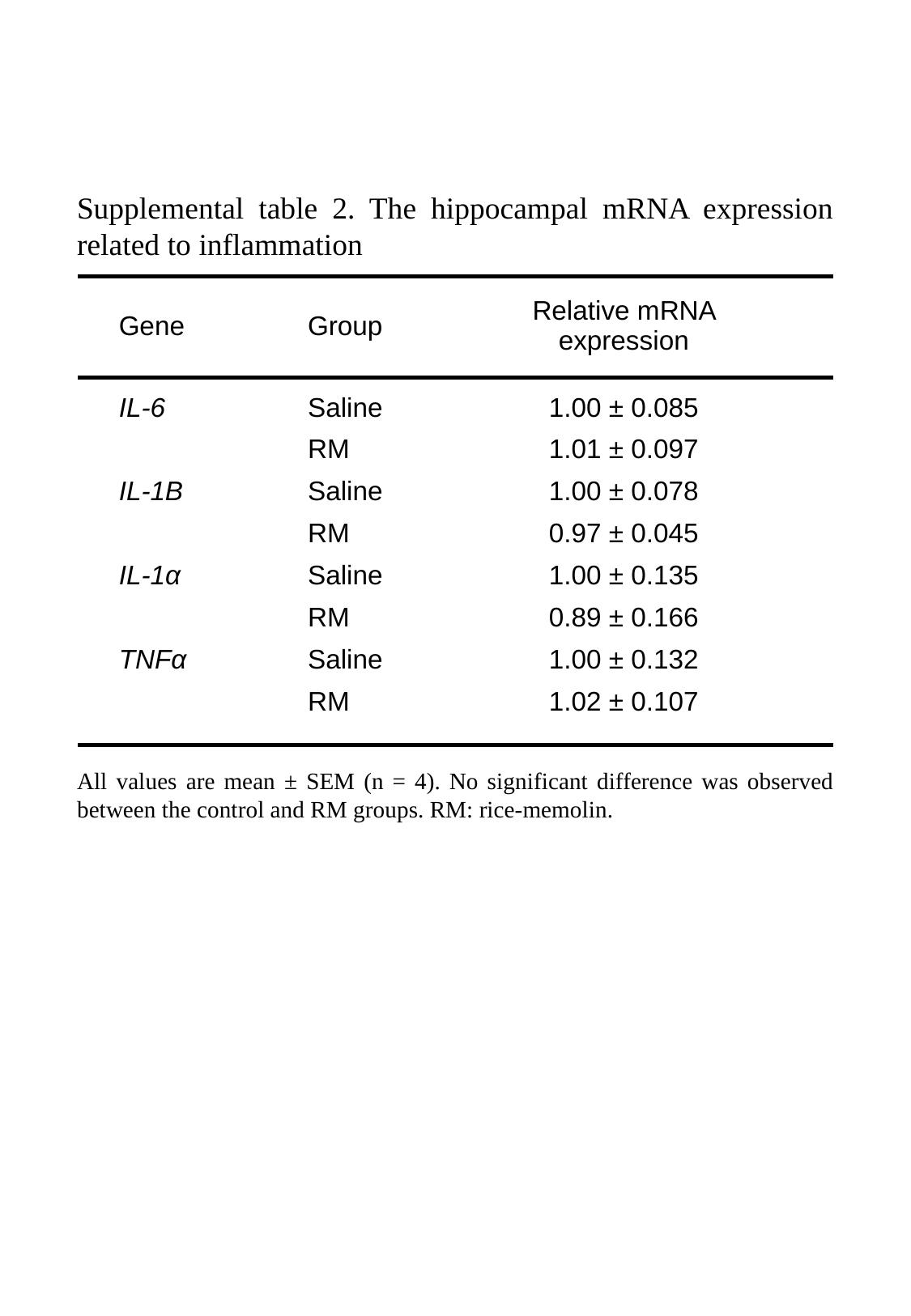

Supplemental table 2. The hippocampal mRNA expression related to inflammation
| Gene | Group | Relative mRNA expression |
| --- | --- | --- |
| IL-6 | Saline | 1.00 ± 0.085 |
| | RM | 1.01 ± 0.097 |
| IL-1B | Saline | 1.00 ± 0.078 |
| | RM | 0.97 ± 0.045 |
| IL-1α | Saline | 1.00 ± 0.135 |
| | RM | 0.89 ± 0.166 |
| TNFα | Saline | 1.00 ± 0.132 |
| | RM | 1.02 ± 0.107 |
All values are mean ± SEM (n = 4). No significant difference was observed between the control and RM groups. RM: rice-memolin.

## Slide 3
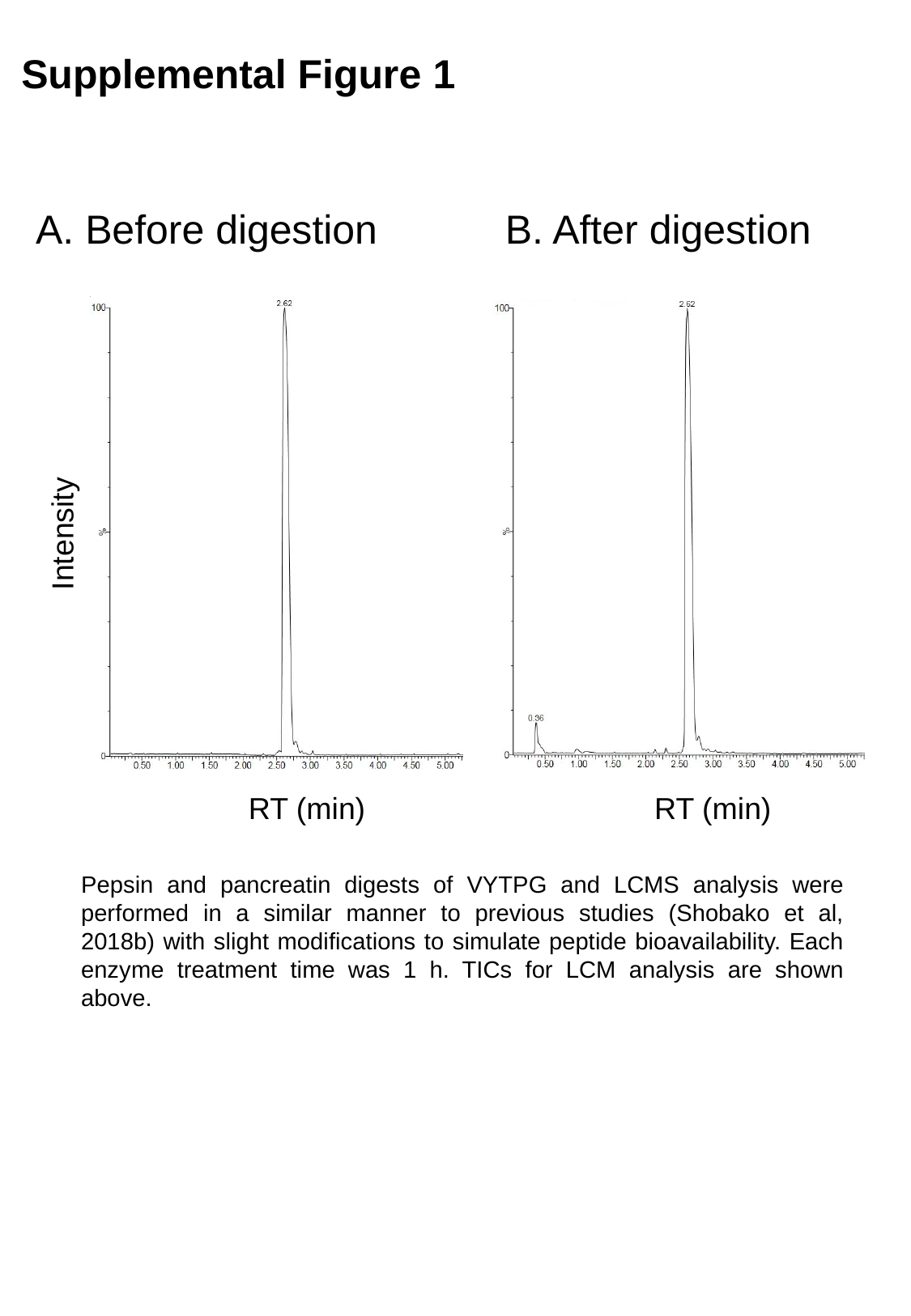

Supplemental Figure 1
A. Before digestion
B. After digestion
Intensity
RT (min)
RT (min)
Pepsin and pancreatin digests of VYTPG and LCMS analysis were performed in a similar manner to previous studies (Shobako et al, 2018b) with slight modifications to simulate peptide bioavailability. Each enzyme treatment time was 1 h. TICs for LCM analysis are shown above.
